# Pancreatic Cancer Chemoresistance to Gemcitabine

**DOI:** 10.3390/cancers9110157

**Published:** 2017-11-16

**Authors:** Manoj Amrutkar, Ivar P. Gladhaug

**Affiliations:** 1 Department of Pharmacology, Institute of Clinical Medicine, University of Oslo, PO Box 1057 Blindern, 0316 Oslo, Norway; 2 Department of Hepato-Pancreato-Biliary Surgery, Institute of Clinical Medicine, University of Oslo, PO Box 1171 Blindern, 0318 Oslo, Norway; i.p.gladhaug@medisin.uio.no; 3 Department of Hepato-Pancreato-Biliary Surgery, Oslo University Hospital Rikshospitalet, PO Box 4950 Nydalen, 0424 Oslo, Norway

**Keywords:** pancreatic ductal adenocarcinoma, gemcitabine, chemoresistance, tumor stroma

## Abstract

Pancreatic ductal adenocarcinoma (PDAC), commonly referred to as pancreatic cancer, ranks among the leading causes of cancer-related deaths in the Western world due to disease presentation at an advanced stage, early metastasis and generally a very limited response to chemotherapy or radiotherapy. Gemcitabine remains a cornerstone of PDAC treatment in all stages of the disease despite suboptimal clinical effects primarily caused by molecular mechanisms limiting its cellular uptake and activation and overall efficacy, as well as the development of chemoresistance within weeks of treatment initiation. To circumvent gemcitabine resistance in PDAC, several novel therapeutic approaches, including chemical modifications of the gemcitabine molecule generating numerous new prodrugs, as well as new entrapment designs of gemcitabine in colloidal systems such as nanoparticles and liposomes, are currently being investigated. Many of these approaches are reported to be more efficient than the parent gemcitabine molecule when tested in cellular systems and in vivo in murine tumor model systems; however, although promising, their translation to clinical use is still in a very early phase. This review discusses gemcitabine metabolism, activation and chemoresistance entities in the gemcitabine cytotoxicity pathway and provides an overview of approaches to override chemoresistance in pancreatic cancer.

## 1. Introduction

Pancreatic cancer ranks fourth among the leading causes of cancer-related mortality in the Western world and is expected to surpass breast cancer, prostate cancer and colorectal cancer to become the second leading cause of cancer-related deaths after lung cancer by 2030 [1]. According to the American Cancer Society estimates, in 2017, about 53,670 people (27,970 men and 25,700 women) will be diagnosed with pancreatic cancer and about 43,090 people (22,300 men and 20,790 women) will die of the disease in the USA alone [2]. The most recent Surveillance, Epidemiology, and End Results (SEER) database estimates an overall five-year survival rate of merely 8.2% (years 2007–2013) for all stages of pancreatic cancer, which is among the lowest of all solid cancer types [3].

Almost 90% of all pancreatic malignancies are pancreatic ductal adenocarcinomas (PDAC). Despite decades of extensive work towards improvement in diagnostic techniques, surgical procedures and chemotherapy, the overall prognosis of PDAC patients remains extremely poor [4,5]. The progression of PDAC from histologically-distinct premalignant lesions to invasive carcinoma is paralleled by successive aggregation of genetic mutations [6,7]. Almost all patients with well-established PDAC carry at least one or more of the four known frequently-mutated driver genes, the oncogene K-RAS and the tumor suppressors CDKN2A, TP53 and SMAD4/DPC4 [7]. Most common risk factors associated with pancreatic cancer include tobacco smoking, family history, chronic pancreatitis, obesity, diabetes and occupational hazards [6]. Due to lack of effective screening methods for early detection and few specific early symptoms, the large majority of PDAC patients are diagnosed with advanced or metastatic disease for whom efficient chemotherapy is mostly lacking [8]. While surgery offers the only potential curative treatment, only 15–20 percent of patients have resectable disease at the time of diagnosis, and even in patients for whom surgical resections are performed, most patients develop disease recurrence within a year [9]. Underlying causes for these dismal results include poor efficacy of treatment modalities as adjuncts to surgery, undetected micro-metastases and development of cellular drug resistance. 

Gemcitabine (also known as dFdC: 2′,2′-difluorodeoxycytidine), originally used for its antiviral effects [10], has been widely used as an anti-cancer chemotherapeutic agent for various solid tumors and currently in certain lymphomas [11]. Since 1997, when Burris et al. showed that gemcitabine was superior to fluorouracil (5-FU) with respect to overall survival (OS), performance status and pain control, gemcitabine has become the standard treatment choice for locally advanced and metastatic pancreatic cancer [12]. Although the effect on survival was merely modest (5.65 months vs 4.41 months), it is noteworthy that the clinical benefit response (CBR) of gemcitabine was more profound, almost five-fold higher, compared to 5-FU (23.8% vs. 4.8%) [12]. The failure of translating good clinical response into relative survival benefits could at least partly be attributed to poor penetration of drug into the hypo-vascularized, dense tumor stroma [13] and to subsequent, within weeks of treatment, development of gemcitabine chemoresistance in initially sensitive tumors [14].

Unlike many other cancer types, the presence of an extensive dense fibrous stroma is a hallmark of pancreatic cancer. This excessive amount of scar tissue (also known as desmoplasia) that surrounds the malignant epithelial cells may account for up to 90% of the total tumor volume [13,15]. The stromal microenvironment surrounding the cancer cells is not a passive bystander, but rather acts as an active contributor to tumor growth and dissemination [16,17]. Desmoplastic stroma predominantly consists of a rich extracellular matrix (ECM) containing cancer-associated fibroblasts (CAFs), inflammatory cells, small blood vessels and a variety of cytokines and growth factors [13,15]. All these components of the stroma interact closely with the malignant cells and offer potential therapeutic targets [18].

The stromal architecture has been postulated to create a physical barrier for drug delivery of gemcitabine and other therapeutic molecules, which has sparked a new era of stromal depletion approaches [19,20]. Since these approaches have so far generally failed to translate into significant clinical benefits, the concept of stroma acting as a physical barrier to chemotherapeutic agents has recently been challenged [21,22]. However, the biochemical crosstalk between stroma and tumor elements is complex, and the actual role of the stroma in development of chemoresistance in pancreatic cancer remains a matter of debate. Various preclinical and clinical studies have reported correlations between that the cellular levels of entities of the gemcitabine metabolism pathway and clinical responses to gemcitabine, suggesting that gemcitabine metabolism rather than biophysical properties matter the most [23,24,25,26]. Despite its broad use, the mechanisms underlying the progression of resistance to gemcitabine still lack clear explanations. Various cell signaling pathways, transcriptional factors and enzymes involved in the nucleosides metabolism contribute to the chemoresistance in PDAC, many of which have been extensively reviewed elsewhere [27,28]. Herein, we focus primarily on the progression of pancreatic cancer chemoresistance linked to alterations in entities associated with the gemcitabine metabolism pathway.

## 2. Chemoresistance in Pancreatic Cancer

Development of tumor resistance to gemcitabine critically limits the efficacy of this cornerstone of pancreatic cancer chemotherapy. Chemoresistance can be broadly classified into two categories: intrinsic (de novo or innate) resistance and acquired resistance [29]. In general, intrinsic resistance refers to the situation when chemotherapy is ineffective from the start of treatment due to patient genetic factors, whereas acquired resistance develops only after a certain time of exposure of tumor cells to anticancer drugs, due to genetic or epigenetic alterations in the cancer cells. In acquired resistance, tumor cells might show drug sensitivity in the beginning of treatment, but continued treatment ultimately leads to refractoriness to chemotherapy [30]. Although pancreatic cancer cells are more susceptible to gemcitabine compared to other anticancer agents, most patients develop resistance within weeks of treatment initiation, leading to poor survival.

Several signaling pathways controlling both intrinsic and acquired resistance in pancreatic cancer have been identified [31]. Pathways regulating both tumor and stromal microenvironment are not necessarily directly linked to gemcitabine cytotoxicity mechanisms; however, they play critical roles in the development of chemoresistance. Signaling pathways regulating growth, proliferation, differentiation, apoptosis, invasion and angiogenesis, such as Akt, epidermal growth factor receptor (EGFR), Notch, mitogen-activated protein kinases (MAPK), nuclear factor (NF)-κB and Sonic Hedgehog (SHH) pathways, appear to, directly or indirectly, impact pancreatic tumor chemosensitivity. Furthermore, cells with an epithelial-to-mesenchymal transition (EMT) phenotype and cancer stem cells seem to be involved in chemoresistance in pancreatic cancer [31].

### 2.1. Desmoplastic Stroma in Chemoresistance

Notably, PDAC treatment shortcomings have been increasingly ascribed to insufficient drug delivery due to decreased microvascularity and stroma-induced chemoresistance. Of the various cell types involved in pancreatic cancer desmoplasia, CAFs are the main fibrosis-producing cells [17]. Generally, pancreatic CAFs are master secretors of both soluble and insoluble factors, which form the specific stromal features that facilitate chemoresistance via physical barriers, as well as transformation of tumor cells and paracrine crosstalk [30]. Pancreatic CAFs are considered to originate from different cellular sources, particularly quiescent pancreatic stellate cells (PSCs), but probably also quiescent resident fibroblasts and bone marrow-derived mesenchymal stem cells (MSCs) [17]. In PDAC, PSCs are activated through multiple activation pathways including platelet-derived growth factor (PDGF), transforming growth factor (TGF)-β, tumor necrosis factor (TNF)-α and interleukins (IL)-1, -6 and -10 [17,32]. Activated PSCs play decisive roles in the desmoplastic reaction and ECM remodeling in PDAC, via secretion of factors such as collagen-type I, matrix metalloproteinases (MMPs) and tissue inhibitors of metalloproteinases (TIMPs) [33,34]. In addition, the multiple CAF stimulated signals in cancer cells are often redundant to specific target(s) of a selected chemotherapy; hence, effective therapy must typically overcome several obstacles at different levels [35,36]. 

Loss of TP53 function inducing activation of JAK2-STAT3 signaling has been shown to promote alterations of tumor stroma, tumor growth and chemoresistance to gemcitabine in mice models [37]. It was also shown that PDAC patients with lower levels of phosphorylated STAT3 and functional TP53 had significantly longer survival compared with patients with high levels of phosphorylated STAT3 and TP53 mutation [37]. Pharmacological inhibition of JAK2 or STAT3 activation in mice lacking TP53 function resulted in reduced fibrosis and number of PSCs in the stroma [37]. Compared to mice treated with control agents, mice treated with a combination of gemcitabine and JAK2 inhibitor formed small-sized tumors and survived longer, and notably, the tumor stroma contained fewer activated PSCs and displayed alterations in collagen production and organization [37].

Another modulator of stromal cells’ derived chemoresistance is the SDF-1α/CXCR4 axis, in which PSCs promote resistance to gemcitabine via paracrine SDF-1α/CXCR4 signaling-induced activation of FAK-AKT and ERK1/2 signaling pathways with subsequent upregulation of IL-6 in cancer cells [38]. Thus, inhibition of PSC interaction with PANC-1 cells via SDF-1α/CXCR4 signaling may provide a promising therapeutic strategy for overcoming stroma-induced gemcitabine resistance in pancreatic cancer [38]. In α-smooth muscle actin (α-SMA)-positive CAFs from human PDAC, inhibition of the mTOR/4E-BP1 protein synthesis pathway abolished CAF-triggered chemoresistance [39]. The combination of gemcitabine with the multi-receptor sst1 somatostatin analogue SOM230, which inhibits the mTOR/4E-BP1 pathway, reduced tumor growth and resistance to gemcitabine in mice xenograft of human PDAC [39].

### 2.2. Stromal Barrier or Drug Metabolism?

The abundant fibrotic stroma of pancreatic tumors has been widely considered as a physical barrier to delivery of gemcitabine and other chemotherapeutic agents to tumor cells, thereby contributing to treatment failure. Consequently, various preclinical approaches for stromal depletion and reversal of vessel compression have been developed and explored. For example, in various genetically-engineered mouse tumor models, pharmacologically-induced stroma remodeling [40,41] or enzyme-mediated degradation of stromal hyaluronan and collagen [42,43], resulted in increased drug delivery and responses to gemcitabine treatment. However, genetically-targeted ablation of stromal fibroblasts in mice tumors appeared to unleash a more aggressive disease with accelerated tumor growth, reduced survival and abolished response to gemcitabine [44,45]. Other studies have indicated that T-cell-mediated immune therapy has beneficial effects despite remodeling of the stoma, including fibroblast depletion [46,47]. Thus, disruption of the stromal barrier to increase drug delivery is not the only factor that increases the anti-tumor response of gemcitabine, and furthermore, increased drug delivery may be most effective when various intra-tumoral survival cues are concomitantly targeted. 

Enhanced drug delivery does not necessarily imply that the anticancer agents are metabolically available and active against tumor cells. For example, systemic and intra-tumoral elevation of gemcitabine in murine models, obtained by co-treatment with a pharmacological inhibitor of gemcitabine inactivation (tetrahydrouridine), did not affect overall tumor growth or apoptotic rate in primary tumors [48]. Furthermore, a recent study by Hessmann et al. reports that fibroblast drug scavenging increases intra-tumoral accumulation of gemcitabine; however, active gemcitabine is entrapped within CAFs and PSCs of the tumor stroma, thus making it unavailable for tumor cells [49]. This study suggests that targeting the metabolic program in CAFs may thus be a promising strategy to enhance the anti-proliferative effects of gemcitabine in PDAC. In addition, this study also provides an alternative explanation for the failure of gemcitabine response in PDAC and challenges the paradigm of a biophysical stroma barrier for gemcitabine delivery. 

## 3. Gemcitabine Pharmacology

### 3.1. Chemical Structure and Properties

**Figure 1:**
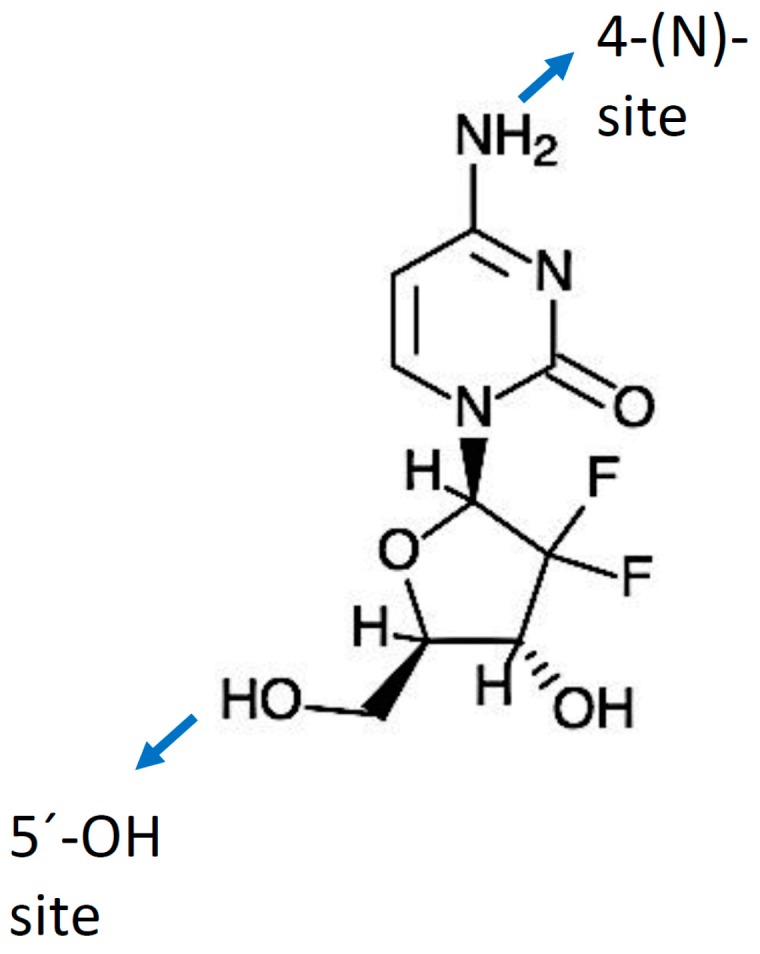
Gemcitabine molecule and its modification sites (4-(*N*) and 5′-OH).

Gemcitabine (dFdC) is a deoxycytidine nucleoside analog whose anti-proliferative properties are dependent on several inhibitory actions on DNA synthesis, blocking cell cycle progression at the G1/S-phase boundary [50]. In comparison with cytosine arabinoside (Ara-C), the first clinically-useful nucleoside analog, gemcitabine has several unique properties and a specific spectrum of activity [51,52]. Distinctive features of gemcitabine in relation to the cellular pharmacology, metabolism and mechanisms of action arise from the structural difference between the fluorine substituents on the second position of the furanose ring of gemcitabine (Figure 1) [53].

### 3.2. Transport, Metabolism and Mechanism of Action 

**Figure 2:**
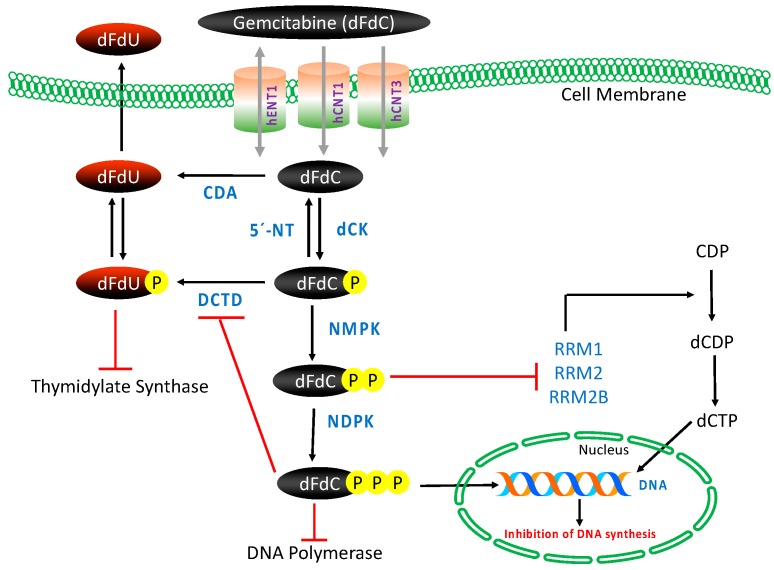
Gemcitabine: transport, intracellular activation/deactivation and mechanism of action. CDA: cytidine deaminase, dCK: deoxycytidine kinase, DCTD: deoxycytidylate deaminase, dFdC: 2′,2′-difluorodeoxycytidine, dFdU: 2′,2′-difluorodeoxyuridine, hENTs and hCNTs: human nucleoside transporters, NDPK: nucleoside diphosphate kinase, NMPK: nucleoside monophosphate kinase, RR(M1/M2), ribonucleotide reductase, 5′-NT: 5′-nucleotidase.

Gemcitabine requires intracellular phosphorylation in order to exert its effects after intracellular uptake (Figure 2); thus, the presence of nucleoside transport activity is a prerequisite for cell growth inhibition and its clinical efficacy [53,54]. The molecule is hydrophilic in nature and thus transported into cells by various human nucleoside transporters (NTs), which include the solute carrier SLC28 family of cation-dependent human concentrative nucleoside transporters (hCNTs) and the solute carrier SLC29 family of energy-independent, human equilibrative nucleoside transporters (hENTs). The hCNTs family members are sodium-dependent symporters that mediate unidirectional transport of nucleosides into cells, while hENTs mediate bidirectional nucleoside transport across biological membranes down a concentration gradient [55]. Gemcitabine is known to be transported into cells by five NTs, hCNT1, hCNT2, hCNT3, as well as hENT1 and hENT2. However, kinetic studies of human cell lines have demonstrated that intracellular uptake of dFdC is mediated primarily by hENT1 (SLC29A1) and, to some extent, by hENT2 (SLC29A2), hCNT1 (SLC28A1) and hCNT3 (SLC28A3) [53,56]. 

Once inside the cell, gemcitabine is phosphorylated into gemcitabine monophosphate (dFdCMP) by deoxycytidine kinase (dCK), and subsequently phosphorylated to gemcitabine diphosphate (dFdCDP) by pyrimidine nucleoside monophosphate kinase (NMPK, also known as UMP/CMP) and gemcitabine triphosphate (dFdCTP) by nucleoside diphosphate kinase (NDPK) [50,53]. The major cellular metabolite of gemcitabine, dFdCTP, acts as a competitive substrate of deoxycytidine triphosphate (dCTP). This allows dFdCTP to be incorporated into DNA during replication, thus inhibiting chain elongation of DNA and causing cell death by apoptosis. The process of `masked chain termination´ appears to lock gemcitabine into DNA. In this process, dFdCTP is incorporated at the end of the elongated DNA strand; once deoxynucleotide is added, the DNA polymerases are unable to proceed, and proof-reading exonucleases are unable to remove gemcitabine nucleotide from this penultimate position [50,53,57].

In this intracellular activation pathway, dCK-mediated phosphorylation of dFdC to dFdCMP is considered the rate-limiting step for subsequent production of active metabolites of gemcitabine [58]. Most of the administered gemcitabine is however inactivated by rapid deamination induced by cytidine deaminase (CDA), thus producing high concentrations of the less active metabolite gemcitabine: 2′,2′-difluorodeoxyuridine (dFdU) [59]. Phosphorylated metabolites of gemcitabine are reduced by cellular 5′-nucleotidase (5′-NT), and the monophosphate form dFdCMP is also converted and inactivated by deoxycytidylate deaminase (DCTD) into 2′-deoxy-2′,2′-difluorouridine monophosphate (dFdUMP) [60,61].

Gemcitabine also possesses a unique mechanism to enhance its own activation, termed “self-potentiation”. The gemcitabine metabolite dFdCDP inhibits ribonucleoside reductase (RR), an enzyme regulating DNA biosynthesis via controlling the formation of nucleoside triphosphates (NTPs). RR converts CDP to dCDP, and its inhibition leads to reduced cellular concentration of the competing dCTP pool necessary for DNA synthesis, thus facilitating incorporation of dFdCTP into DNA. In addition, dFdCTP and intracellular reduction of dCTP suppress the inactivation of dFdCMP by DCTD, the activity of which requires a sufficient level of active dCTP [53,60,62].

## 4. Gemcitabine Metabolism-Associated Entities in Chemoresistance

Chemoresistance entities associated with gemcitabine metabolism pathways include drug transporters, activating and inactivating enzymes and competitive substrates to active metabolites. Their roles in pancreatic cancer chemoresistance are discussed here (Table 1).

### 4.1. Nucleoside Transporters 

Pancreatic cancer patients with low tumor expression of nucleoside transporters show significantly worse survival compared to patients with high hENT1 and hCNT3 levels following gemcitabine treatment [69,70,71,72]. It has also been shown that cells deficient of NTs are resistant to gemcitabine-induced cytotoxicity [56]. hENT1 and hCNT1 levels correlate with gemcitabine sensitivity and OS, making them good predictive markers for gemcitabine response in pancreatic cancer patients as high hENT1 expression is associated with longer OS and disease-free survival (DFS) of pancreatic cancer patients [63,69,71,73]. hENT1 levels are correlated with gemcitabine response in vitro as upregulation of hENT1 enhances the cytotoxic effect of gemcitabine, while loss of hENT1 results in developing resistance to gemcitabine [56,74]. Reduced hENT1 expression leading to limited intracellular influx of gemcitabine is a well-established phenomenon [56]. These observations might suggest that enhanced hENT1 expression in tumor cells could possibly give additional survival benefits after gemcitabine treatment. Recently, some progress has been made in this direction since pretreatment with thymidylate synthase (TS) inhibitors resulted in enhanced hENT1 expression in tumor cells [75]. However, additional studies are necessary to confirm whether this effect can be translated to in vivo experimental models and ultimately to clinical settings. Gemcitabine is also transported into cells partly via hENT2 [76]; however, marked differences in the kinetics of gemcitabine transport by recombinant hENT1 and hENT2 in *Xenopus* oocytes have been observed. hENT1 transports gemcitabine with high affinity and low capacity, while hENT2 transports gemcitabine with low affinity and high capacity (*K*_m_ for gemcitabine: hENT1 < hENT2) [76].

In contrast to hENT1, the role of hCNT1 in the regulation of gemcitabine-induced cytotoxicity in pancreatic cancer has not been well described. Some data indicate that hCNT1 expression is frequently reduced in pancreatic tumors and pancreatic cancer cell lines compared with normal pancreas and pancreatic ductal epithelial cells [64]. Gemcitabine-resistant pancreatic cancer cells exhibit relatively limited, cell cycle-dependent hCNT1 expression and gemcitabine influx. However, it has been shown that pharmacological inhibition of hCNT1 degradation could moderately increase the cellular gemcitabine transport, suggesting a possible mechanism to augment gemcitabine transport and chemosensitization [64,77].

Several novel mechanisms regulating NTs activity have been reported. The transmembrane glycoprotein mucin 4 (MUC4) is aberrantly expressed in pancreatic cancer and associated with increased invasiveness and inversely correlated with prognosis [78]. MUC4 expressing pancreatic cancer cells exhibit greater resistance to gemcitabine than MUC4 negative cells, through activation of anti-apoptotic pathways, thereby promoting cell survival [79]. Furthermore, it has been shown that MUC4 inhibits hCNT1 expression via the NF-κB pathway, whereas inhibition of MUC4 induced increased levels of both hCNT1 and hCNT3, leading to enhanced gemcitabine sensitivity [80]. MUC4 and its membrane partner the oncogenic receptor ErbB2 interact physically in pancreatic cancer cells [81], and silencing of ErbB2 results in enhanced gemcitabine sensitivity via upregulation of hCNT1 and hCNT3 expression [82]. 

Recently, it has been reported that in the PDAC microenvironment, PSCs are a source of the matricellular cysteine-rich angiogenic inducer 61 (CYR61) protein, which in co-culture models with pancreatic cancer cell lines induces chemoresistance in tumor cells by downregulating NTs [83]. Computed tomography (CT)-derived transport properties’ measurement in patients with resectable PDAC tumors showed significant inter-patient and intra-tumoral heterogeneity of gemcitabine incorporation into DNA despite similar intravascular pharmacokinetics [84]. It is noteworthy that stromal content correlated with gemcitabine DNA incorporation only after accounting for levels of hENT1 [85].

### 4.2. Deoxycytidine Kinase 

Deoxycytidine kinase is the main rate-limiting enzyme of intracellular activation and metabolism of gemcitabine, and its expression generally corresponds to the degree of gemcitabine resistance in pancreatic cancer patients [70,86]. A clear correlation between dCK activity and gemcitabine sensitivity has been demonstrated in both human and murine xenografts. Human pancreatic cancer cell lines with acquired resistance to gemcitabine demonstrate frequent inactivation of dCK. Knockdown of dCK resulted in gemcitabine resistance, while overexpression of dCK into gemcitabine-resistant cell lines resulted in restored gemcitabine sensitivity [65,87,88]. Immunohistochemically, low expression of dCK correlated with both reduced OS and old patient age, suggesting age-related epigenetic regulation of the dCK gene in pancreatic cancer patients [89]. Sequencing of the entire dCK coding sequence of pancreatic cancer cell lines, as well as tumor tissue from patients with disease progression while on gemcitabine treatment did not identify any mutations, indicating that genetic alterations or coding polymorphisms of dCK are not a common mechanism for intrinsic resistance to gemcitabine in pancreatic cancer. However, the levels of pretreatment dCK tumor protein content have been shown to be correlated with OS after gemcitabine treatment, and its expression is considered to be stable even after development of resistance to gemcitabine [89]. This suggests that determination of dCK immune-labelling prior to initiation of gemcitabine therapy may improve OS by identifying patients that are less likely to respond to this treatment. 

Tumors from PDAC patients show elevated levels of the RNA-binding stress-response protein Hu antigen R (HuR, encoded by the ELAVL1 gene) compared to normal pancreas. HuR associates with, and enhances, the expression and activity of dCK. Upon exposure to gemcitabine, HuR translocates from the nucleus to a cytoplasmic localization in pancreatic cancer cells and, through its dCK regulating activity, sensitizes the cells to gemcitabine [90,91]. Targeted inhibition of HuR resulted in impairment of malignant characteristics of PDAC in both cancer cells and murine xenografts [92]. dCK protein expression positively correlates with HuR protein levels and efficacy of gemcitabine, accordingly overexpression of HuR elevates, while silencing of HuR reduces dCK protein expression, conferring corresponding gemcitabine responses in pancreatic cancer cells. Notably, for patients receiving adjuvant treatment with gemcitabine, patients with low cytoplasmic HuR expression are reported to be at a seven-fold increased risk of cancer death compared to patients with high HuR levels [90,93]. 

### 4.3. Cytidine Deaminase

Major inactivation of gemcitabine occurs through cytidine deaminase-induced deamination of dFdC to dFdU via removal of the -NH_2_ group from pyrimidine [59]. The uracil metabolite dFdU is not a substrate for pyrimidine nucleoside phosphorylases; hence, it is degraded and excreted out of the cells. Clinically, dFdU is the only metabolite of gemcitabine found in the urine of gemcitabine-treated patients [94]. CDA expression has been correlated with OS in pancreatic cancer patients, as well as preclinical responses to gemcitabine [23,24,25,26]. Several in vitro studies report that upregulation of CDA results in gemcitabine resistance, whereas loss of CDA restores gemcitabine sensitivity [26,88,95]. This suggests that alteration of CDA levels in PDAC tumors might provide a mechanism to increase gemcitabine sensitivity; however, further studies are necessary to substantiate this assumption.

### 4.4. 5′-Nucletidase 

Cellular 5′-nucleotidase opposes the activity of dCK via dephosphorylation of dFdCMP, resulting in partial inactivation of gemcitabine by preventing formation of dFdCTP; hence, 5′-NT levels could be one of the factors affecting the clinical outcome of gemcitabine therapy. Analysis of gemcitabine metabolites in murine pancreas cancer models revealed accumulation of large amounts of the active metabolite dFdCTP concomitant with reduced amounts of the inactive metabolite dFdU in stromal fibroblasts (PSCs) when compared to epithelial cancer cells, and this observation was linked to the low levels of intracellular 5′-NT in the stromal cells [49]. It has also been shown that the cellular phenotype of the gemcitabine-resistant leukemia K562 cell line was associated with enhanced cellular 5′-NT activity [96] and that overexpression of cytosolic 5′-nucleotidase I (5′-NT-I/cN-I) confers resistance to several pyrimidine analogs [97]. In malignant cells obtained from non-small cell lung cancer patients treated with gemcitabine-based chemotherapy, it was reported that only the level of 5′-NT-I was correlated with OS [98]. Notably, the primary focus concerning 5′-NT has been its use as a putative indicator of prognosis, rather than understanding its role in pancreatic cancer gemcitabine resistance.

### 4.5. Ribonucleotide Reductase 

Ribonucleotide reductase consists of two subunits, M1 and M2. The M1 subunit RRM1 possesses a binding site for enzyme regulation, while the M2 subunit RRM2 is involved in RR activity. RR is a rate-limiting enzyme of the DNA synthesis pathway, mainly responsible for conversion of ribonucleotides to dNTPs, which is essential for DNA polymerization and repair. Inhibition of RR reduces the endogenous dNTP pool, thus reducing competition and indirectly facilitating dFdCTP incorporation into DNA. dFdCDP-induced inhibition of RR is the most important mechanism involved in the potentiation of gemcitabine activity [60,62]. In pancreatic cancer patients treated with gemcitabine, RRM1 levels inversely correlate with OS where high expression of RRM1 is associated with poor survival, suggesting an important role for RRM1 in intrinsic resistance to gemcitabine [70,71,99]. Notably, in PANC-1 pancreatic cancer cells, overexpression of both RRM1 and RRM2 was found to be a necessary requirement for development of resistance to gemcitabine [66]. However, in gemcitabine-resistant MIA PaCa-2 cancer cells, knockdown of RRM1 could completely overcome the gemcitabine resistance. A synergistic effect between gemcitabine and hydroxyurea, an RR inhibitor, on gemcitabine-resistant cancer cells was also observed [100]. Further evidence for the importance of RRM1 in maintaining gemcitabine resistance in pancreatic cancer cells has been provided by studies of the MEK1/2 inhibitor pimasertib, which reduced RRM1 protein expression via post-translational modifications concomitantly with increased sensitivity to gemcitabine [79]. This also suggests that combining MEK inhibitors with gemcitabine is a potential strategy to improve the efficacy of gemcitabine in patients with pancreatic cancer.

Inhibition of RRM2 induced by dFdCDP, leading to a reduced dCTP pool, has distinct effects on nuclear DNA, i.e., facilitating the incorporation of dFdCTP into replicating DNA. It has been demonstrated that overexpression of RRM2 results in reduced gemcitabine sensitivity while RRM2 knockdown leads to enhanced gemcitabine sensitivity in pancreatic cancer cells and in human pancreatic cancer xenografts in mice models [81]. Furthermore, in clinical studies, mRNA levels of RRM2 inversely correlate with OS in gemcitabine-treated pancreatic cancer patients [58,99,101]. Of all the cellular mechanisms mediating gemcitabine transport and metabolism, the most studied include downregulation of the nucleoside transporter hENT1 and the rate-limiting enzyme dCK, as well as upregulation of RRM1/RRM2. In an effort to combine the underlying gene expressions in a model for predicting gemcitabine sensitivity, it was demonstrated that a ratio of mRNA expression of [(hENT1 × dCK)/(RRM1 × RRM2)] decreased progressively with the development of acquired resistance to gemcitabine in pancreatic cancer cells [70]. Thus, this ratio may be a useful predictive marker for the efficacy of gemcitabine chemotherapy in pancreatic cancer patients.

### 4.6. Thymidylate Synthase 

Thymidylate synthase is a folate-dependent enzyme that catalyzes the conversion of 2′-deoxyuridine-5′-monophosphate (dUMP) into 2′-deoxythymidine-5′-monophosphate (dTMP), an essential precursor for DNA synthesis, and its inhibition controls depletion of intracellular nucleotide pools, making this enzyme a critical target in cancer chemotherapy [67]. Inhibition of TS also activates hENT1, thereby enhancing the responsiveness of gemcitabine [102]. A randomized phase II study, GEMSAP, in advanced pancreatic cancer patients treated with combined gemcitabine and S-1 (an oral prodrug of 5-FU) showed enhanced progression-free survival (PFS), as well as OS, compared to treatment with gemcitabine alone [103]. The deaminated metabolite of gemcitabine, dFdUMP, may serve as either a substrate or an inhibitor of TS. Loss of TS expression decreased resistance to gemcitabine in pancreatic cell lines. Furthermore, the protein expression of TS in tumors from pancreatic cancer patients has been positively correlated with resistance to gemcitabine and inversely related to DFS in these patients [104]. Downregulation of TS upon gemcitabine exposure was observed in gemcitabine-resistant pancreatic cancer cells, while TS was upregulated in gemcitabine-sensitive pancreatic cancer cells [68]. The TS expression also provides an alternative source of substrate for DNA synthesis and positively correlates with gemcitabine resistance and shortened patient survival [104].

## 5. Potential Ways to Improve Gemcitabine Delivery and Efficacy

Gemcitabine is well known to be metabolically unstable and possesses a low therapeutic efficacy particularly due to CDA-induced deamination and rapid clearance in the bloodstream. Furthermore, gemcitabine has poor membrane permeability and depends on NTs for intracellular uptake, thus limiting its desired cytotoxic effects in the target cells. To compensate for these limitations, usually a high dose of gemcitabine (about 1000 mg/m^2^) is administered, which in turn generates severe side effects such as breathlessness, neutropenia, nausea and kidney failure [61]. Modification of the gemcitabine molecule aimed at improved bioavailability and efficacy, as well as novel strategies for improved drug delivery are being explored to extend the use of gemcitabine in pancreatic cancer therapy. Modifications of the gemcitabine molecule are mainly based on prodrug and nanocarrier approaches, which are discussed below (Table 2). 

### 5.1. Prodrug Approach

A “prodrug” is a biologically inactive form of a parent drug molecule exhibiting better delivery properties than the parent drug and that requires an enzymatic or chemical transformation within the body to release the active drug entity [61]. Different chemical modifications at two sites of the gemcitabine molecule (i.e., (4-(*N*)-position and 5′-position) have been developed yielding various prodrugs of gemcitabine with better activity and efficacy compared to native gemcitabine [61]. Chemical modifier entities at the 4-(*N*)-position of the gemcitabine molecule include various acyl derivatives (valeroyl, heptanoyl, lauroyl and stearoyl) [123,124], polyethylene glycol (PEG), vitamin E succinate (VES), 1,1′,2-tris-nor-squalenoic acid (squalene) and valproic acid. Modifier entities at the 5′-OH position of gemcitabine include cardiolipin, elaidic acid and a series of phosphoramidates [61,125]. The resulting conjugates of gemcitabine have demonstrated their potential to improve clinical outcomes of traditional gemcitabine-based therapy [61,125].

#### 5.1.1. Modifications at the 5′-OH Position 

NeoPharm synthesized a novel gemcitabine-cardiolipin conjugate (NEO6002), which displayed higher anti-tumor activity in BxPC-3 human pancreatic tumor models in mice [114] and enhanced uptake and efficacy by prolonged release of gemcitabine in various cancer cell lines including BxPC-3 [115]. The cytotoxicity induced by NEO6002 in BxPC-3 cells was shown to be independent of NTs activity, and in BxPC-3 xenograft bearing mice, NEO6002 was observed to be less toxic compared to free gemcitabine at equimolar dosages [115]. Furthermore, growth inhibition induced by NEO6002 in BxPC-3 xenografts was significantly higher compared to free gemcitabine (52% vs. 32% after 50 days of treatment) [115]. Another lipophilic prodrug, gemcitabine-elaidic acid conjugate CP-4126 (CO-101), has been shown to be absorbed by cancer cells independent of hENT1 levels [116,117]. CP-4126 possesses a broad spectrum of anti-proliferative activity both in vitro and in a wide range of human tumor models in vivo, including pancreas [116]. Gemcitabine and CP-4126 were observed to be equally effective in chemoresistant cancer cell lines and various xenograft models, whereas both were found to be ineffective in cells lacking dCK activity. In mice models, CP-4126 could be administered orally, and its efficacy was maintained in NT-inhibited cells, while improved in tumors with low or no hENT1 expression [61,116]. However, in a randomized phase II study, CO-101 (CP-4126) was not superior to gemcitabine with respect to survival in patients with metastatic PDAC with low hENT1 expression. In addition, hENT1 expression in metastatic tumors was not predictive of the response to CP-4126, and there was no correlation between hENT1 expression and gemcitabine sensitivity [126]. In a phase I and pharmacokinetic study, CP-4126 was found to be well tolerable with a comparable toxicity profile to gemcitabine [127].

To bypass the obligatory rate-limiting phosphorylation step in the gemcitabine activation pathway, a variant of the monophosphate form, the phosphoramidate prodrug of gemcitabine, has been developed [118]. The aim of this modification was to overcome the gemcitabine resistance in dCK-deficient tumors by delivering dFdCMP intracellularly via passive diffusion. This prodrug was observed to be about four-fold more effective than gemcitabine in dCK-deficient variants of leukemic and ovarian cancer cell lines (CEM-dCK and AG600). Dipyridamole-induced inhibition of NTs activity did not diminish the prodrug’s activity in the dCK variants. Furthermore, its anti-tumor activity was mediated by cell entry via nucleoside transport [118]. This approach was extended to synthesis and screening of a series of phosphoramidate prodrugs of gemcitabine, such as NUC-1031 6f, in which a phosphoramidate ProTide moiety has been added [119]. Compared to gemcitabine, NUC-1031 6f prodrug activation was significantly less dependent on NTs and dCK. In addition, it was resistant to CDA-mediated degradation and directly generated dFdCMP intracellularly. In pancreatic cancer xenograft mouse models, it showed a significant reduction in tumor volume compared to gemcitabine [120]. ProTide 6f is currently under clinical development in a phase I/II study [120].

#### 5.1.2. Other Modifications

To further improve permeability and enzymatic stability, gemcitabine prodrugs with D- and L-configuration amino acids were synthesized. Prodrugs containing D-amino acid gemcitabine showed higher plasma concentration and superior enzymatic stability compared to L-amino acid gemcitabine prodrugs. Both prodrugs were more potent than parent gemcitabine in AsPC-1 pancreatic cancer cells [121]. Likewise, in another report, the dipeptide prodrugs of gemcitabine showed significantly higher uptake and superior anti-proliferative ability compared to the parent drug in the pancreatic cancer cell lines AsPC-1 and PANC-1 [122]. 

### 5.2. Nano-Carrier Approach

**Figure 3:**
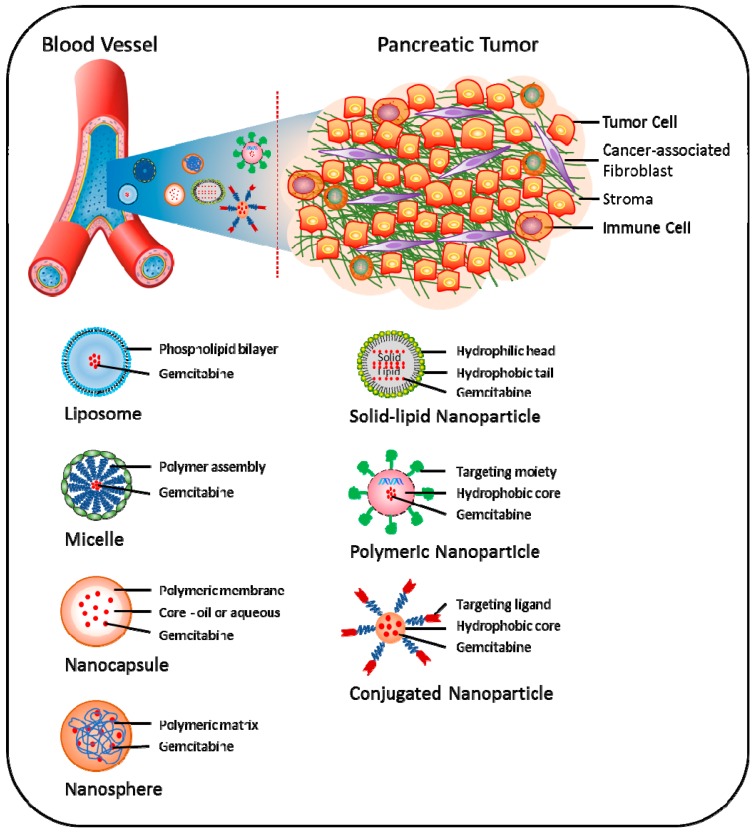
Approaches being explored to target pancreatic tumor using gemcitabine-based nanomedicines. Different nanocarriers, such as liposome, micelle, nanocapsule, nanosphere, polymeric nanoparticle, solid-lipid nanoparticle and conjugated-nanoparticle, have been developed for effective delivery of gemcitabine to pancreatic tumor cells. Strategies for the efficacy of gemcitabine using nanomedicines include, but are not limited to increased drug availability via protection from first-pass metabolism, passive accumulation at the tumor site due to the enhanced permeability and retention (EPR) effect, as well as targeted delivery of gemcitabine to tumor cells

Recently, the concept of nanoparticle (NP)-based drug delivery of gemcitabine has been introduced as a promising new tool to overcome various pathological and pharmacological barriers and thereby attenuate chemoresistance in pancreatic cancer (Figure 3) [128]. Using this approach, increased drug concentration in tumor tissues can be obtained via enhanced permeability and retention (EPR) effects, i.e., NPs accumulate more in tumor than in non-normal tissues [129]. Nano-carrier approaches have demonstrated enhanced efficacy both in vitro and in vivo; however, its potential clinical use is still under development [130].

Combined use of lipophilic modifications and nanoscale drug delivery is an attractive approach to overcome the challenges of gemcitabine delivery to cancer cells. For example, polymeric micelles (PM) containing 4-(*N*)-stearoyl gemcitabine (GemC18) and its self-assembled NPs have shown higher cytotoxicity than gemcitabine and GemC18 alone [105]. Notably, GemC18 NPs showed better cellular uptake and cytotoxicity than the PM formulation in the pancreatic cancer cell lines AsPC-1 and PANC-1 [105]. In mice models for pancreatic cancer, GemC18 NPs showed better anti-tumor properties compared to free gemcitabine, while PEGylated GemC18 NPs showed significantly enhanced blood circulation time and accumulation into tumor tissues [106]. Furthermore, PEG2000-hydrazone-C18 conjugate (PHC-2) micelles containing GemC18 have been shown to inhibit RRM1 expression and enhance the levels of dFdCTP in gemcitabine-resistant cancer cells [107]. GemC18 was also shown to overcome RRM1-induced resistance to gemcitabine upon incorporation into solid lipid nanoparticles (SLNPs) [131,132,133]. Compared with free gemcitabine, VES-dFdC nanocapsules have shown enhanced cellular uptake and intracellular controlled release of drug and superior growth inhibition activity in BxPC-3 cells [110]. The cellular uptake of these nanocapsules by BxPC-3 cells was up to seventy-times higher than that of native gemcitabine during the first 1.5 h of incubation; however, the stability of these nanocapsules was limited [110]. To enhance the stability of VES-dFdC nanocapsules and increase its concentration in PBS or isotonic solution, co-assembled nanoformulations of D-α-tocopheryl polyethylene glycol succinate (TPGS)/VES-dFdC have been developed. These nanoformulations exhibited 4.7-times higher anti-tumor activity in nude mice with pre-established BxPC-3 tumors, compared to native gemcitabine [112].

To bypass the rapid inactivation of gemcitabine, a novel squalene (SQ)-dFdC NP formulation was shown to overcome gemcitabine resistance in BxPc-3, Capan-1 and PANC-1 cells [113]. Furthermore, it was demonstrated that SQ-dFdC NPs were able to partially circumvent the three important determinants of resistance to gemcitabine, i.e., downregulation of hENT1, downregulation and inactivation of dCK and deaminase-induced deactivation [134]. This strategy has been further extended with the development of the monophosphate prodrug SQ-dFdCMP nano-assemblies, which displayed higher anti-proliferative and cytotoxic effects in chemoresistant pancreatic cancer cells and significantly decreased tumor growth in a human pancreatic MIA PaCa-2 carcinoma xenograft model in mice, compared to free gemcitabine [135]. These effects were associated with a reduction of Ki-67 antigen expression and induction of apoptosis mediated by caspase-3 activation in tumor cells [135].

Gemcitabine molecules are either encapsulated or adsorbed in NPs, resulting in reduced pre-systemic metabolism, lower dosage demands and sustained release. For example, compared to free gemcitabine, its encapsulation into the albumin NPs generated sustained release profiles, as well as improved anti-tumor activity in BxPC-3 cells [136,137]. For targeted delivery of gemcitabine to pancreatic cancer cells, modified NPs with the addition of monoclonal antibodies as a targeting moiety have been utilized. For example, Herceptin (HER2)-conjugated chitosan NPs loaded with gemcitabine have shown increased anti-proliferative activity along with enhanced S-phase arrest in pancreatic cancer cells, compared with free gemcitabine. Notably, these NPs were efficiently taken up by the cells, and prolonged intracellular retention was observed [138]. Similarly, administration of gold NPs loaded with the anti-EGFR antibody cetuximab and gemcitabine was shown to inhibit both pancreatic cancer cell proliferation in vitro and orthotopic pancreatic tumor growth in vivo [139].

To enhance the half-life of gemcitabine, the design and development of a methacrylate-based monomer conjugate of gemcitabine, which was polymerized by reversible addition-fragmentation chain transfer (RAFT) polymerization, was recently reported. The polymer conjugate NPs exhibited significant cytotoxicity in pancreatic cancer cells. Both monomer and polymer conjugates displayed prolonged activity; however, IC_50_ for both was higher compared to the parent drug [140]. To improve the delivery, encapsulation of gemcitabine in polymeric nanocapsules was achieved via the inverse miniemulsion periphery RAFT polymerization (IMEPP) approach. Gemcitabine-loaded nanocapsules showed two-fold higher cytotoxicity in AsPC-1 cells compare to free gemcitabine [141]. 

The nano-carrier approach has been further extended to combination therapies. For example, the combination of gemcitabine and a polymeric encapsulated NP formulation polymeric nanoparticle-encapsulated curcumin (NanoCurc) was shown to enhance tumor growth inhibition, abolish systemic metastases and reduce activation of NF-κB in a pancreatic cancer mouse xenograft model, compared to either agent alone [142]. A novel drug design involving covalent pre-conjugation of two or more therapeutic agents through hydrolysable linkers enables loading of multiple drugs onto the same nanocarrier [143]. A dual-drug nanocarrier delivery system of paclitaxel-gemcitabine conjugates significantly improved cytotoxicity in pancreatic cancer cells as compared to the free drug conjugates [143]. 

Compared to gemcitabine alone, the combination of nanoparticle bound paclitaxel (nab-paclitaxel) plus gemcitabine treatment in pancreatic cancer patients has shown higher response rates (23% vs. 7%), median OS (8.5 months vs. 6.7 months) and PFS (5.5 months vs. 3.7 months) [144]. In mouse models, the combination treatment of gemcitabine plus nab-paclitaxel has shown increased gemcitabine concentration in plasma and tumor tissues [145]. The increased intra-tumoral levels and activity of gemcitabine were due to marked reduction in the CDA levels, the primary gemcitabine-metabolizing enzyme. The reduction of CDA is attributed to nab-paclitaxel, as in vitro experiments in mouse pancreatic adenocarcinoma cells had demonstrated that paclitaxel actually reduced CDA protein levels in cultured cells [23,146]. This suggests that higher response rate and survival benefits observed in pancreatic cancer patients treated with the combination of gemcitabine plus nab-paclitaxel are possibly linked to reduced CDA levels, reduced deamination of gemcitabine and, thereby, resultant enhanced activity. Several ongoing clinical trials are examining the effects of gemcitabine-based combination therapies in pancreatic cancer patients (Table 3). 

## 6. Conclusions and Future Directions

Since the 1997 report by Burris et al. [12], gemcitabine has remained the standard of care for locally advanced and metastatic PDAC despite only marginal effects on patient survival. In the following two decades, gemcitabine alone or in combination with fluoropyrimidine, platinum analogues or the EGFR inhibitor erlotinib has represented the most commonly-used front-line treatment options for PDAC therapy. At present, this is gradually shifting with recent positive results from phase III clinical studies that established the new first-line treatment choices of FOLFIRINOX (5-FU, leucovorin, irinotecan, oxaliplatin) and the doublet of gemcitabine + nab-paclitaxel [144,147]. This extended the panel of available chemotherapies, for the first time achieving significant survival benefits for patients with metastatic PDAC; however, increased rates of toxicity often limit frequent clinical use of both regimens. Despite the treatment advances, gemcitabine remains a cornerstone of neo-adjuvant, adjuvant, as well as palliative therapy for advanced PDAC.

To achieve efficient therapeutic regimens, the clinician primarily relies on balancing anti-tumor effects and the toxicity profile to normal tissues. Considering the rapid deamination of gemcitabine to its inactive metabolite dFdU and the need for undergoing series of phosphorylation for its activation, gemcitabine poses severe limitations as a drug of choice, particularly toxicity due to high and repeated dosages and the development of chemoresistance. The chemoresistance mechanisms include a lack of transporters, a lack of activating enzymes and/or enhanced levels of deactivating enzymes. Numerous chemical modifications and encapsulation designs of the gemcitabine molecule have been proposed to overcome the resistance mechanisms. Typically, prodrugs that protect the amine function (4-(*N*)-position) block CDA-induced inactivation of gemcitabine, while entrapment of gemcitabine into colloidal systems such as liposomes and NPs improve its pharmacokinetic profile, leading to improved bio-distribution and site-specific drug delivery.

The prominent dense desmoplastic tumor stroma and sparse, collapsed vasculature, which are characteristic features of PDAC, contribute distinctly to the formation of the chemoresistant phenotype [148]. It has been shown in multiple studies that the tumor stroma promotes tumor progression, invasion, metastasis and chemoresistance in PDAC [149]. Recently, a paradigm shift has taken place in the approach towards understanding the development of chemoresistance to gemcitabine in pancreatic cancer, with a number of recent studies focusing on stroma-induced epithelial regulation of chemosensitivity [150]. Numerous phase I–III clinical trials are currently investigating stromal depletion in order to enhance angiogenesis and effective drug delivery in pancreatic tumors [151]. However, considering the tumor diversity, it is imperative to understand the heterogeneity of pancreatic tumors and how chemotherapeutic drugs exert their efficacy.

Although chemoresistance entities of the gemcitabine pathway are well characterized, numerous other cellular and tumoral determinants pose obstacles in overcoming chemoresistance in PDAC. Thus, pursuing the targets of interest to improve chemotherapy efficacy remains challenging. To overcome the limitations inherent in gemcitabine transport mechanisms, activation and overall clinical response, significant efforts are currently underway. Despite more than two decades of clinical use and obvious therapeutic challenges, gemcitabine alone or in combinations remains a cornerstone in PDAC chemotherapy. In efforts to improve PDAC chemotherapy, it is of profound importance to understand the fate of gemcitabine and its metabolites and the relative contribution of stromal and epithelial tumor components to the chemoresistant phenotype.

## Figures and Tables

**Table 1:** Gemcitabine metabolism-associated chemoresistance entities in pancreatic cancer.

Target Entity	Role in Gemcitabine Metabolism	Resistance Pattern	Impact on Progression of Chemoresistance to Gemcitabine	References
Nucleoside transporters	Drug transport	Downregulation	Level of hEN1, hCNT1 and hCNT3 are correlative of resistance to gemcitabine	[63,64]
Deoxycytidine kinase	Intracellular drug activation	Downregulation	Reduced levels of dCK are linked with acquired resistance to gemcitabine	[65]
Cytidine deaminase	Drug inactivation	Upregulation	CDA induced deamination causes degradation and excretion of gemcitabine	[26]
Ribonucleotide reductase	Competition in DNA synthesis	Upregulation	RR mediates DNA synthesis via generation of dCTPs	[66]
Thymidylate synthase	Competition in DNA synthesis	Upregulation	Regulation of early stages of DNA biosynthesis, activation of salvage pathway	[67,68]

**Table 2:** Modifications of the gemcitabine molecule and their outcome in pancreatic cancer.

Position	Target Moiety	Prodrug	Experimental Model	Outcomes	References
4-(*N*)	Acyl derivative (stearoyl)	GemC18	AsPC-1 and PANC-1 cells, murine BxPC-3 tumor xenografts	Inhibition of RRM1 and increased dFdCTP levels, enhanced anti-tumor activity	[105,106,107]
	Polyethylene glycol (PEG)	PEG–NHS	MIA PaCa-2 and PANC-1 cells	Prolonged circulation in murine plasma, improved cytotoxicity and apoptosis	[108]
		PEG-PCC	MIA PaCa-2 and L3.6 cells, murine MIA PaCa-2 tumor xenografts	High anti-tumor activity and increased apoptosis	[109]
	Vitamin E succinate (VES)	VES-dFdC	BxPC-3 cells	High anti-tumor activity, enhanced cellular uptake	[110]
	D-ɑ-tocopheryl PEG succinate	TPGS/VES-dFdC	BxPC-3 cells and murine BxPC-3 tumor xenografts	High anti-tumor activity, resistant to CDA induced deamination and superior cytotoxicity	[111,112]
	1,1′,2-tris-nor-squalenoic acid (squalene)	SQ-dFdC/SQ-dFdCMP	BxPC-3, Capan-1, PANC-1 cells; murine BxPC-3, MIA PaCa-2 and PANC-1 tumor xenografts	High anti-proliferative and cytotoxic effects, reduced tumor growth and prolonged survival	[113]
5′-OH	Cardiolipin	NEO6002	BxPC-3 cells and murine BxPC-3 tumor xenografts	High cytotoxicity independent of NT activity and high tumor growth inhibition	[114,115]
	Elaidic acid	CP-4126 (CO-101)	Murine MIA PaCa-2, PANC-1 tumor xenografts	Transport independent of hENT1, equally effective to gemcitabine	[116,117]
	Phosphoramidate	Mono-phosphate	Cell lines with dCK-deficient variants: AG600 and CEM-dCK	~4-fold more effective than gemcitabine	[118]
	Phosphoramidate ProTide	NUC-1031 (ProTide 6f)	BxPC-3, MIA PaCa-2, PANC-1 cells; murine MIA PaCa-2, tumor xenografts	Resistant to CDA mediated deamination and directly generates dFdCMP intracellularly; reduced tumor volume	[119,120]
Other	D-amino modifications	-	AsPC-1 cells	High plasma concentration superior enzymatic stability	[121]
	Dipeptide monoester prodrugs	-	PANC-1, and AsPC-1 cells	Enhanced uptake and anti-proliferation activity	[122]

**Table 3:** Ongoing clinical trials for gemcitabine-based combination therapies in pancreatic cancer.

Drug Combination	ClinicalTrials.gov Identifier	Disease Condition (Pancreatic Cancer)	Study Phase
Gemcitabine + Abraxane	NCT02043730	Stage II	II
Gemcitabine + Erlotinib	NCT02154737	Locally advanced	I
Gemcitabine + SRA737	NCT02797977	Locally advanced	I
Gemcitabine + Cisplatin, +/−Veliparib	NCT01585805	Metastatic	II
Gemcitabine + Capecitabine	NCT02919787	Locally advanced	II
Gemcitabine + S-1	NCT02131493	Locally advanced	II
Gemcitabine + Metformin	NCT02005419	Stage IA, IB, IIA, IIB	II
Gemcitabine + All-trans retinoic acid (ATRA)	NCT03307148	Locally advanced or metastatic	IB

## Abbreviations

The following abbreviations are used in this manuscript:

5′-NT	5′-nucleosidase
CAFs	cancer-associated fibroblasts
CDA	cytidine deaminase
CNT	concentrative nucleoside transporter
dCK	deoxycytidine kinase
DCTD	deoxycytidylate deaminase
dCTP	deoxycytidine triphosphate
dFdC	2′,2′-difluorodeoxycytidine
dFdU	2′,2′- difluorodeoxyuridine
DFS	disease-free survival
ECM	extracellular matrix
ENT	equilibrative nucleoside transporter
NT	nucleoside transporters
NP	nanoparticle
OS	overall survival
PDAC	pancreatic ductal adenocarcinoma
PEG	polyethylene glycol
PSCs	pancreatic stellate cells
RR	ribonucleotide reductase
